# Fresh Start, a postpartum weight loss intervention for diverse low-income women: design and methods for a randomized clinical trial

**DOI:** 10.1186/s12889-016-3520-0

**Published:** 2016-09-09

**Authors:** Milagros C. Rosal, Christina F. Haughton, Barbara B. Estabrook, Monica L. Wang, Germán Chiriboga, Oahn H. T. Nguyen, Sharina D. Person, Stephenie C. Lemon

**Affiliations:** 1 Division of Preventive and Behavioral Medicine, University of Massachusetts Medical School, 55 Lake Avenue North, Worcester, MA 01655-0002 USA; 2 Department of Quantitative Health Sciences, University of Massachusetts Medical School, 55 Lake Avenue North, Worcester, MA 01655 USA; 3 Boston University School of Public Health, 801 Massachusetts Avenue, Boston, MA 02215 USA; 4 Harvard School of Public Health, 677 Huntington Avenue, Boston, MA 02215 USA; 5 Family Health Center of Worcester, Inc, 26 Queen Street, Worcester, MA 01610 USA

**Keywords:** Postpartum weight loss, Obesity, Women, Health disparities, Randomized clinical trial, Narrative interventions

## Abstract

**Background:**

Overweight and obesity are prevalent among young women and are greater among minority and low-income women. The postpartum period is critical in women’s weight trajectories as many women do not lose their pregnancy weight, and others lose some and then plateau or experience weight gain. Excess weight puts women at greater risk of chronic disease and thus weight loss in the postpartum period may be key to the long-term health of young women. This paper describes the design and methods of a randomized clinical trial of Fresh Start, an innovative narrative-based group intervention aimed at promoting postpartum weight loss among low-income, diverse women.

**Methods/design:**

Study participants were recruited from the five sites of the Women, Infants and Children (WIC) program in central Massachusetts. Participants were English-speaking, age ≥ 18 years, 6 weeks to 6 months postpartum, with a body mass index (BMI) ≥ 27 kg/m^2^. The Fresh Start postpartum weight loss intervention, adapted from the Diabetes Prevention Program (DPP) in collaboration with WIC staff and clients, consisted of an 8-week group-based curriculum followed by nine monthly telephone calls. It included a narrative component (i.e., storytelling), group discussions, print materials and access to exercise facilities. The study is a two-arm randomized controlled trial. The control condition included print materials and access to exercise facilities. In-person assessments were conducted at baseline and at 6 and 12 months following the eight-week intervention phase.

**Discussion:**

The Fresh Start intervention translated key elements of an evidence-based weight loss protocol into a format that is hypothesized to be relevant, acceptable and effective for the target audience of low-SES postpartum women. This novel intervention was developed in collaboration with WIC to be sustainable within the context of its clinics, which reach approximately 9 million individuals per year across the U.S. via 10,000 clinics.

**Trial registration:**

clinicaltrials.gov NCT02176915. Registered 25 June 2014.

## Background

Approximately 60 % of women age 20 to 39 are overweight or obese, with 32 % considered obese [1]. Furthermore, minority, less educated and socioeconomically disadvantaged young women have a greater prevalence of obesity than more affluent non-Latino white women [2–5]. Overweight and obesity are risk factors for diabetes, cardiovascular disease and some cancers, and weight loss and weight gain prevention during young adulthood are associated with decreased risk of chronic disease [6–11].

The postpartum period is critical and influences weight trajectories, and in turn disease risk, of low- SES women over the lifecourse [12, 13]. Although most women lose some pregnancy weight during the first 6 weeks postpartum, many either plateau or gain weight thereafter [12, 14]. On average, women retain 3 kg of weight per pregnancy 10 years after giving birth [15], with excess weight retained after childbirth often being centrally distributed [16]. Racial/ethnic minority and low-SES women are at greater risk of postpartum weight retention [14–18]. These groups also have the highest pregnancy rates, conferring additional risk.

The Women Infants and Children (WIC) program is an important setting in which to deliver weight loss interventions as it offers tremendous reach and sustainability for low- SES postpartum women. WIC, a federal- and state-funded program, provides free nutritional health services to more than 9 million individuals via 10,000 clinics across the US [19]. Previous studies have tested a range of weight loss interventions among postpartum WIC clients. The interventions varied in approach including a DVD-based intervention that was complemented by peer support group teleconferences [20], a peer-led group intervention that was compared to a self-guided intervention [21], a promatora-led group intervention designed to enhance social support for physical activity [22], and technology-based interventions [23]. Except for a pilot study by Herring and colleagues [23] which showed promising results after a 14-week intervention, each study demonstrated minimal or no significant weight loss and other limitations that include lack of systematic implementation of evidence-based weight loss strategies (i.e., limited focus on creating an energy deficit; non-systematic use of evidence-based weight loss strategies), limited attention to opportunities for physical activity among this population, and poor intervention attendance. Studies also reported high attrition rates. No systematically translated evidence-based interventions currently exist for promoting weight loss among low-SES women.

To reduce disparities in postpartum weight retention, innovative approaches to weight loss interventions addressing a multitude of factors faced by these women (e.g., socioeconomic, literacy, psychosocial, interpersonal, contextual, cultural) are needed. At present there is a need for studies of adapted evidence-based weight loss programs like the Diabetes Prevention Program Lifestyle Intervention (DPP) [10], which include core elements of behavioral self-management in a way that is sensitive to the circumstances, needs and preferences of the low-income new mothers. Incorporation of narrative intervention components, that is, communication of intervention messages through stories into group-based weight loss interventions, is a promising approach. Narrative interventions are an increasingly popular and effective component of health behavior change programs and are hypothesized to be more emotionally and intellectually engaging than didactic approaches [24]. Narratives are used to increase cognitive processing of health messages [25] by tapping deep cultural structures (i.e., the worldview of a cultural group) [26], providing surrogate social connections, and addressing emotional and existential issues [25, 27]. Narratives are a potentially powerful component in interventions with racial/ethnic minority groups and individuals with low literacy levels. To date, no previous study has tested narrative components in the context of a group-based weight loss intervention.

### Study objectives

The goal of this study was to test the effectiveness and implementation of Fresh Start, a postpartum weight loss intervention designed to be culturally and literacy appropriate for diverse, low-SES postpartum women, and for potential sustainability within WIC clinics. Specifically, the lifestyle intervention from the DPP was modified to be briefer, relevant to diverse low-income postpartum women, with narrative components integrated into the intervention protocol. This paper describes the design and methods for the randomized clinical trial of Fresh Start.

## Methods/Design

### Study design

This 2-arm randomized controlled trial targeted enrollment of postpartum women between 6 weeks and 6 months after childbirth and compared the narrative-based Fresh Start intervention (*n* = 60) to a condition consisting of print materials and access to exercise facilities (*n* = 60). In-person measures were collected at baseline and at 6 and 12 months following the 8-week intensive intervention phase. The study protocol received approval from the Institutional Review Board of the University of Massachusetts Medical School and the Board of Directors of the Family Health Center of Worcester.

### Study site and participants

Study participants were 139 clients served by 5 WIC clinics in Worcester, MA. Eligibility criteria included: 1) childbirth in the previous 6 weeks to 6 months; 2) age 18 and over; 3) body mass index (BMI) ≥ 27 kg/m^2^; 4) English-speaking; and 5) obstetric provider approval for participation in the diet and physical activity components of the intervention and weight loss. Exclusions included: 1) being unable or unwilling to give informed consent; 2) being pregnant or planning to become pregnant within the following 24 months; 3) having a psychiatric illness which limits ability to participate; 4) taking a medication that causes weight changes; 5) having no telephone; and 6) planning to move out of the area within the study period.

### Screening and recruitment

During routine visits, WIC providers completed a checklist of selected study eligibility criteria (i.e., BMI, date of delivery, age ≥ 18 years, English speaking, ability to consent) to identify potentially eligible clients based on chart information and gave a study fact sheet to the women and inquired about their interest in learning more about the study if they were potentially eligible. Interested women were asked to provide their contact information. Potentially eligible and interested women were then contacted by the study recruiter via telephone to explain the study further and ascertain interest. If verbal consent was provided, additional eligibility screening was conducted (i.e., comfortable speaking in English, plans to reside in the study area over the following 1.5 years, not pregnant or planning to become pregnant, no self-reported health reasons precluding physical activity or calorie restriction, able to attend sessions if randomized to the group intervention condition). Potentially eligible women were scheduled for a study visit at which women were consented for confirmation of BMI eligibility. Eligible and interested women were required to provide written consent for study participation prior to completing the baseline assessment measures. Upon receipt of health care provider approval, women were then randomized to the intervention or the control condition. Baseline characteristics of the study sample are shown in Table 1.

**Table 1:** Sample baseline characteristics (*n* = 139)

Characteristics	N or mean	Percentage or standard deviation
Age	28.19	5.73
Marital status		
Never married	61	44.2 %
Married/Living as married	72	52.17 %
Separated /Divorced	5	3.62 %
Race		
Hispanic/Latina	50	36.23 %
Black or African American	33	23.91 %
Native African	3	2.17 %
Asian	4	2.90 %
American Indian/Alaska Native	3	2.17 %
White	45	32.61 %
Foreign born	43	31.15 %
Education		
< HS	16	11.94 %
High school graduate/GED	45	33.58 %
Some college/2-year degree	53	39.55 %
≥ 4-year college graduate	20	14.93 %
Number of children	2.18	1.35
Perceived sufficiency of income		
More than need	0	
Just enough	67	48.55 %
Not enough	62	44.93 %
Doesn’t know	9	6.52 %
Body Mass Index (BMI)	34.72	6.46
Waist circumference (inches)	37.39	3.91
Perceived health status		
Excellent	14	10.07 %
Very good	41	29.50 %
Good	64	46.04 %
Fair	16	11.51 %
Poor	4	2.88 %

### Intervention development

#### DPP adaptation

The Fresh Start intervention was adapted from the DPP Lifestyle Intervention in collaboration with the WIC program in Worcester, MA and the WIC state office at the MA Department of Public Health. The initial process for adaptation has been described elsewhere [28]. Briefly, the intervention was guided by Social Cognitive Theory (SCT) [29] and the Social Ecological Model (EM) [30], and used an intervention adaptation framework involving: 1) building a partnership with the WIC partners with the goal of developing an informed postpartum weight loss intervention for WIC clients; 2) Understanding the target setting (i.e., organizational structure and potential for integrating an intervention into routine practice) through a process of key informant interviews with WIC supervisors and nutritionists at four sites; 3) Understanding the target population through program information on overweight/obesity prevalence, key informants interviews with WIC staff and with clients; 4) Developing the intervention derived from the DPP, with format, channels, and messages adapted based on data gathered through the previously outlined steps; 5) Refining the intervention for clarity, acceptability, and perceived usefulness using key informant interviews. The intervention was subsequently pilot tested and evaluated through quantitative and qualitative assessment [28].

#### Narrative component

The narrative component intended to address challenges observed in the pilot study: 1) Limited intervention attendance among participants, 2) Recommendation among pilot participants for including guest speakers in the intervention groups, and 3) Difficulty among nutritionists leading the pilot groups in achieving fidelity to protocol delivery principles (i.e., motivational interviewing principles; eliciting socio-cultural experiences related to diet, activity and weight loss; steering discussions that facilitate modeling of behavior change among participants), appearing more comfortable simply “teaching” information. Drawing from our previous experiences with narrative interventions [31–33], this intervention added a narrative component (i.e., storytelling videos) to address these limitations through identification with storytellers to maximize participant engagement and modeling opportunities, presentation of a variety of perspectives on how to implement behavior change strategies, and to assist the nutritionists in eliciting culturally relevant group discussions.

Development of the narrative component of the intervention included the following:

1. Story development/focus groups were used to identify narratives relevant to the intervention and “star” storytellers (i.e., women with compelling stories matching the intervention topics). Story development groups are a type of focus group in which the group facilitator invited participants to tell their stories about their experiences with postpartum weight loss using a prompted story development guide. Recruitment for story development groups included reaching out to women who participated in the pilot intervention in addition to recruitment of postpartum women in community settings serving low-income women (*n* = 25).
2. Video-recorded interviews with the identified “star” storytellers (*n* = 15) used individualized interview guides designed to help women re-tell their stories in greater depth, with particular emphasis on topics and theoretical constructs targeted by the intervention.
3. Transcribing and coding interviews was done to identify and rate unique story units (noting start and stop timestamps for a complete story and/or response to each interview guide question). Stories with relevant content were coded for theoretical constructs (self-efficacy, outcome expectancies, knowledge, positive and negative reinforcements, behavioral capabilities for self-management, and modeling characteristics) and intervention topics. Coding was conducted independently by two members of the research team (MW, BE) and discrepancies were resolved in consultation with a third member (MCR) until 100 % inter-rater agreement was achieved.
4. Story units were categorized by interviewee and theoretical construct and assigned to specific intervention sessions based on topic. Two members of the team rated the stories assigned to each session based on: level to which the story addressed common issues in the target population (0 = not; 1 = somewhat; 2 = highly) and story completeness/usability and storyteller engagement (scale of 1-5, with 5 being the highest). Stories with the highest ratings and that targeted more than one construct were selected for use in one of 3 pre-planned videos (for sessions 1, 3, and 7), for pre-testing. In a focus group, women from the target community were asked to watch the videos and discuss their reactions, including perceived message, identification with the storyteller, transportation (extent to which the viewer is absorbed into the storyline), elements most useful, and motivation for behavior change based on the story/message. Based on this feedback, the final videos for the sessions were created. To keep the length of the videos under 15 min, the stories were edited by a professional editor to optimize video flow and omit extraneous or distracting footage.

### Intervention format and objectives

The Fresh Start intervention protocol included eight in-person 90-min weekly group sessions (intensive phase) followed by 9 monthly individual telephone follow-up calls. A WIC program nutritionist implemented the group intervention sessions and calls following a detailed provider manual. The nutritionist was trained in behavioral weight loss strategies, principles of motivational interviewing and the use of the Fresh Start intervention tools. A WIC assistant made reminder calls prior to each session, conducted weigh-ins at the beginning of each session and assisted participants with recording and graphing their weight in a program sheet. The weight loss and associated behavioral objectives included weight loss of 1–2 lb per week by working toward a goal of consuming approximately 1500 and 2000 calories for non-breastfeeding and breastfeeding mothers, respectively, and walking 10,000 steps (or equivalent) per day.

The group sessions were interactive and built on one another. Participants were instructed to track their physical activity, diet and weight in between sessions. Sessions began with a review of the participants’ records since the previous session and discussion of successes and challenges to goal achievement, followed by a review of the previous session. A storytelling video relevant to the session topic (topics depicted in Table 2) was then shown, followed by a guided discussion that intended to highlight the women’s experiences regarding the topic being presented. Group discussions were intended to promote modeling opportunities among the women by highlighting similarities among women and exploration of challenges to behavior change and successful strategies to overcome them. Sessions also sought to promote support among participants (i.e., encouragement to exercise together). All sessions concluded with goal-setting.

**Table 2:** Fresh Start intervention session topics

Session #	Topic(s)
1.	Exploring and sharing weight loss motivations; Getting started at being active; Introducing goal setting
2.	Tracking food and beverage intake to make better eating choices
3.	Identifying problem eating habits; Three ways to reduce calories
4.	Reading nutrition labels; Making dietary modifications
5.	Cutting calories on a budget
6.	Taking charge of the environment: Food cues
7.	Taking charge of what’s within you: Talking back to negative thoughts
8.	Dealing with slips; Staying motivated

Supplemental print materials were adapted from the DPP, and included: a workbook with key session topics, and content and graphics relevant to young mothers (e.g. a list of weight loss motivations for new mothers, illustrations depicting the concept of energy balance, weekly menus with shopping list; a physical activity/exercise menu); a food and activity guide to facilitate tracking of energy intake and expenditure; goal-setting forms; and self-monitoring sheets for tracking daily weight and energy intake and expenditure. Participants were provided with a scale, measuring cups and spoons, a step counter, and access to the local YWCA fitness facilities through a corporate 12-month membership purchased by the study. Participants were encouraged to attend the sessions with their infants and babysitting was provided on-site for older children. At each session, a healthy 500-calorie meal was provided for participants and their children. At the monthly calls, the nutritionist reviewed the participant’s goal attainment and weight loss progress, explored successes and challenges, reviewed any relevant content from the group sessions, and assisted the participant in setting new goals. No new content, strategies or narratives were introduced during the follow-up phase.

### Comparison condition

The comparison condition was a self-directed intervention in which the Fresh Start print materials were delivered by mail to participants at the outset, along with access to the YWCA via a membership similar to that of the participants in the group intervention. While there is no evidence to support that self-directed or print-based interventions impact weight loss [34, 35], the comparison condition was chosen in order to improve recruitment through the promise of receiving some type of program and to improve retention through maintaining ongoing contact [34, 35].

### Outcome variables and assessments

Table 3 describes the study measures. The primary outcomes were change in weight and BMI. Secondary outcomes were waist circumference, blood pressure, physical activity and diet. Surveys additionally collected information on participant psychosocial and socio-demographic characteristics. Because of threat to contamination, we included a series of questions asking about participants’ potential knowledge of and discussions with other members of the study in the follow-up surveys. Surveys were verbally administered by trained research staff to ensure a higher completion rate given varying literacy levels of participants. In addition, the study examined potential external validity and sustainability of the Fresh Start intervention on measures of reach, adoption, implementation and maintenance, consistent with the RE-AIM framework [36].

**Table 3:** Measurement protocol in the Fresh Start study

Measure	Assessment approach/tool
Weight/BMI	Measured with the individual wearing light clothing and no shoes using portable digital scales and stadiometers
Waist circumference	Measured twice (and averaged) with a non-stretchable measuring tape using standard methodology
Blood pressure	Measured three times over 60–90 min in a standardized manner [42], after several minutes of quiet sitting with a Dinamap XL automated BP monitor [43]
Physical activity	The Pregnancy Physical Activity Questionnaire [44]
Diet and Eating Behaviors	NCI computerized 24 h recall [45], Three-Factor Eating Questionnaire, Weight Behavior Inventory [46]
Other behaviors	Pittsburgh Sleep Quality Index [47], daily weighing, Behavioral Risk Factor Surveillance System tobacco use questions, CAGE alcohol screening questionnaire
Psychosocial	Edinburgh Postnatal Depression Scale [48], Perceived Stress Scale [49], Delaying Gratification Index (food questions), perceived weight status (investigator developed), family social support for weight loss (investigator-developed)
Socio-demographics	Education, income, employment, race/ethnicity, age, marital status, living situation, number of children, smoking, weight history, parity, breastfeeding and chronic conditions.
Reach	Number, percentage and representativeness of women who participate in the study, including recruitment and retention rates and attendance at intervention sessions.
Adoption	Counts of uptake of the intervention by new WIC clinics after the research study, assuming effectiveness
Implementation	*Intervention delivery*: fidelity to intervention delivery using a standardized checklist with comments completed by research staff
	*Costs*: materials costs and time required to deliver both conditions tracked using a log of study-related expenses, which will be summarized by category (e.g. staff time, food costs, materials, etc.)
	*Utilization, or dose received*: participant attendance counts for group and follow-up phone call session attendance; surveys at follow-up sessions for print material use and YWCA use
Maintenance	*WIC clinics program delivery*: maintenance of the intervention beyond the research funding, including where and to whom the intervention was delivered for a period of 2 years
	*Participant behaviors*: weight change at 12 month follow-up assessment

### Participant retention

Strategies used to maximize participant retention included: request for two alternate telephone numbers; use of personalized letters mailed to non-responding participants encouraging them to call the study coordinator to discuss their status; use of motivational messages to re-kindle original motivations to join the study; provision of incentives after each assessment; and written consent from participants’ to update their contact information through the WIC database if needed. To facilitate timely assessments, a tracking system alerted assessors about participants due for follow-up measures.

### Statistical approach and power

Sample size calculations were based on the primary hypothesis that participants in the Fresh Start intervention would demonstrate greater weight loss than participants in the comparison condition at 6 and 12 months follow-up. In our pilot, we observed a weight change of -4.6 lbs (SD = 8.2lbs). Assuming the Fresh Start intervention would yield a similar change of -5.0 lbs and conservatively that the comparison condition would maintain their weight (0 lbs change), a two-sided, two-sample *t*-test of means with alpha = 0.05 and 85 % power indicates that enrolling 50 people per group would allow us to detect the desired difference. Thus, the original target sample size was 120 participants based on power calculations. We exceeded this goal, enrolling 139 participants.

### Planned analyses

Analysis to test the study hypotheses will be conducted in Spring, 2017 when the 12-month follow-up data collection is completed. The longitudinal aspect of the design involves repeated measures at three time points (baseline, and 6 and 12 months post-intensive intervention). To accommodate the correlation between baseline and subsequent time points we plan mixed models regression for primary and secondary analyses. The main predictors of the model include intervention indicator, time points, and the interaction term between intervention indicator and time points. The treatment effects will be evaluated by testing the interaction term of time and the indicator of intervention. We will use exploratory graphical analysis to examine the relationship of the outcome of interest and baseline factors not in balance to determine the functional form of the model fit. Upon determining the best models, we will then include an indicator of the intervention group to estimate and test the effect of the intervention. Model building strategies described by Harrell [37] will be used to build the multivariable models. There is always the chance that some unmeasured confounder could impact on the results. We will estimate the strength of the confounding that would be necessary to eliminate any measured differences between intervention and comparison groups through sensitivity analyses [38, 39]. We will explore site-level effects by estimating random effects models, although we do not anticipate such an effect.

All primary analyses will be conducted on an “intent-to-treat” basis with each patient enrolled in the intervention group analyzed as part of the group, regardless of engagement with the intervention. Additionally, multiple imputation methods will be used to account for those lost at the follow-up sessions.

Planned analyses also include assessing the potential external validity and sustainability of the Fresh Start intervention on measures of reach, adoption, implementation and maintenance, as described in Table 3. Descriptive statistics (i.e. counts, proportions, means) will be used to describe each of these measures.

### Data safety and monitoring plan

A center-wide Data Safety Monitoring Board (DSMB) was convened. Because a DSMB was not required by the funder and The Fresh Start project was deemed to be of low risk to participants, the DSMB was comfortable in leaving oversight of the project to the local IRB, the Administrative Core of the center, and the Internal and External advisory committees. The DSMB did make itself available on an as-needed basis.

## Discussion

Fresh Start translated key elements of an evidence-based weight loss protocol into a format that is hypothesized to be relevant, acceptable and effective for the target audience of low-SES postpartum women. The group-based intervention was developed through a series of formative projects, and pilot tested. It incorporated messages regarding weight loss motivations specific to low-SES postpartum women and their contextual realities, including cultural resources and barriers. Through a narrative component, the intervention integrates social modeling to facilitate change in theoretical constructs related to weight loss, including self-efficacy and outcomes expectations, and cultural tailoring by using video stories of diverse low-SES postpartum women who have made successful steps toward weight loss in circumstances that resemble those of the target population (i.e., weight loss motivations, family circumstances, time and financial limitations).

The project involved stakeholder engagement to design an intervention that has potential to transform practice. To impact public health, weight loss interventions must be widely implemented in real-world settings. The Fresh Start intervention was developed in collaboration with WIC to be sustainable within the context of WIC clinics, which reach approximately 9 million individuals per year across the U.S. via 10,000 clinics [19], and has a mission of influencing lifetime nutrition, health behaviors, and healthy weight among its clients. While postpartum weight loss is consistent with the mission of WIC, the program does not provide weight loss services and there are no prior studies documenting efficacious interventions in the WIC setting.

The few interventions that have been developed specifically to address weight loss in WIC settings have been unsuccessful in achieving weight loss [21, 40]. This study addresses limitations of prior studies [20, 21, 41], including a lack of systematic implementation of evidence-based weight loss strategies, limited attention to opportunities for physical activity among this population, and poor intervention attendance. Fresh Start was designed with real-world implementation in mind. If effective, the Fresh Start intervention has potential to reduce overweight and obesity among diverse, low-SES postpartum women. Our approach to intervention development recognizes that culturally responsive intervention adaptation requires a comprehensive understanding of the culture of the target population as well as the organization that will eventually sustain and disseminate the intervention. Given the wide reach of WIC to low-income mothers, an effective weight management program in the WIC setting has great potential for dissemination.

## Abbreviations

BMI: Body mass index
DPP: Diabetes prevention program
WIC: Women, infants and children program
